# Human Microbiome Acquisition and Bioinformatic Challenges in Metagenomic Studies

**DOI:** 10.3390/ijms19020383

**Published:** 2018-01-27

**Authors:** Valeria D’Argenio

**Affiliations:** 1 CEINGE-Biotecnologie Avanzate, via G. Salvatore 486, 80145 Naples, Italy; dargenio@ceinge.unina.it; Tel.: +39-081-373-7909; 2 Department of Molecular Medicine and Medical Biotechnologies, University of Naples Federico II, via Pansini 5, 80131 Naples, Italy; 3 Task Force on Microbiome Studies, University of Naples Federico II, 80131 Naples, Italy

**Keywords:** human microbiome, 16S rRNA analysis, metagenomics, metrascriptomics, data analysis, bioinformatics

## Abstract

The study of the human microbiome has become a very popular topic. Our microbial counterpart, in fact, appears to play an important role in human physiology and health maintenance. Accordingly, microbiome alterations have been reported in an increasing number of human diseases. Despite the huge amount of data produced to date, less is known on how a microbial dysbiosis effectively contributes to a specific pathology. To fill in this gap, other approaches for microbiome study, more comprehensive than 16S rRNA gene sequencing, i.e., shotgun metagenomics and metatranscriptomics, are becoming more widely used. Methods standardization and the development of specific pipelines for data analysis are required to contribute to and increase our understanding of the human microbiome relationship with health and disease status.

## 1. Introduction

The advent of Next-Generation Sequencing (NGS) technologies has given rise to metagenomic research during the last ten years. Indeed, NGS-based methods have provided the capability of simultaneously analyzing communities of microorganisms without the need of an a priori knowledge of their composition, and overcoming cultivation steps [1,2].

Based on these technological advances, a plethora of studies have been carried out to characterize the microbial communities of specific environments, including human beings [3,4,5,6,7,8,9]. In particular, the mutually beneficial relationship between the microbiome and the human host has been largely investigated [2,10,11,12,13,14]. Just in the last ten years, more than 35,000 scientific reports have been published on metagenomic aspects, about 30,000 of which focus on the human microbiome, and it is easy to predict that this number will progressively increase in a short time.

As already defined elsewhere [2], the human microbiota is the community of microorganisms that lives on the surface and inside the human host (mucosal surfaces, skin, tooth surface, respiratory airways, gut, urogenital system, etc.). In this view, the so-called human holobiont, or superorganism, is the ensemble of human and microbial cells present in a specific ecosystem, whose heterogeneity depends on the body site and on the host’s genetic background [15]. Even if the initial statement assessing that bacterial cells overcome human cells by a ratio of 10:1 has been recently revised [16], tuning it down to a 1:1 ratio, it means that we are made at least for a half of bacteria. Based on these data, it is clear that the human microbiota is associated with physiological functions required for the human host healthy status acquisition and maintenance. Thus, if these functions are impaired, because of a microbial dysbiosis, this could lead to pathological phenomena. According to this statement, microbial alterations have been investigated and identified in a number of diseases [17,18,19,20,21,22,23]. Quantitative and/or qualitative microbial alterations, in fact, not only could play a role in diseases onset, thus increasing our knowledge on diseases pathogenetic mechanisms, but could represent also novel, sometimes not invasive (depending on the samples in which they have to be investigated), diagnostic biomarkers or targets for novel therapeutic approaches aiming at dysbiosis correction. However, despite the initial enthusiasm and the interesting findings reported to date, most studies remain at an observational level, while less is known about the complex interplays among the human host and his microbiome, both in health and, consequently, in specific diseases.

The aim of this paper is to underscore the relevance of the gut microbiome for human physiology and health maintenance. In addition, since the increasing capability of data production imposes new challenges for their accurate analysis, the main bioinformatic tools for metagenomics will be examined.

## 2. Human Microbiome Acquisition and Healthy Status Maintenance

Humans’ relationships with their microbiome begin already before birth. Up to a few years ago, we thought that humans were born sterile and that microbial colonization began immediately at birth, being influenced by the kind of delivery [2,24]. In fact, a central dogma of reproductive medicine assesses that the fetus stays in a sterile environment and that the newborn acquires its own microbiota only after birth [25].

Increasing evidence suggests that bacterial colonization may be initiated in utero [24,25]. Collado et al. studied the microbiome present in the maternal feces, placenta, amniotic fluid, colostrum, meconium, and infant feces collected from women undergoing elective caesarean delivery and their babies [26]. Interestingly, not only they found bacterial communities in all the analyzed samples, but also assessed that the placenta and the amniotic fluid share common features, being characterized by a lower diversity and high levels of Proteobacteria compared to the other maternal and fetal samples. In addition, after 3–4 days of life, the infant fecal microbiome appears to become more similar to that of colostrum [26]. Thus, the progressive human gut microbial colonization probably starts already during prenatal life by a specific microbiota resident in the placenta and amniotic fluid and is then continued at delivery and after birth by the feeding habits. The origins of the placental and meconium microbiota are not well known. Different potential sites (including maternal vagina, mouth, and gut) have been suggested, even if the most probable hypothesis is that different bacteria may have different origins [27,28]. Consequently, the maternal microbiota during pregnancy might influence fetal development. Therefore, a maternal dysbiosis occurring during pregnancy may be silent in the mother, but may have consequences on the fetus and the placenta. A maternal dysbiosis, in fact, could trigger inflammatory reactions and may induce an anomalous immune system activation potentially affecting fetal development or inducing preterm delivery [27,28,29]. These findings suggest that the prenatal microbial “invasion” is not only crucial to modulate fetal development but may also influence the adult healthy status [24,25,26,27,28,29].

After birth, infants are suddenly exposed to the external environment (including surfaces, air, food) and, being a “naive” environment, they are like an attractive pole for bacteria: this process appears to be essential for immune system development [30]. Despite this invasion, it has been noticed that a strong selection occurs according to the body sites to maintain tissue-specific composition [24,25,30,31,32]. Several environmental factors can modify infants’ microbiome composition, mainly depending on maternal habits [2,24,25,30,31,32]. In particular, the effects of the delivery mode on microbiome composition have been investigated [33,34]. It has been described that children born by cesarean section, especially by elective cesarean section, have a reduced microbial richness and a reduced quantity of health-associated bacterial species (i.e., *Lactobacilli*) [33,34,35]. Dominguez-Bello et al. analyzed the microbial composition in the feces of babies born by vaginal and cesarean delivery and compared these samples with the microbial communities present in the maternal mouth, skin, and vagina [34]. First of all, they confirmed the presence of a different microbiota in infant fecal samples depending on the delivery mode. Next, a diversity analysis revealed a tight clusterization between maternal skin and the gut microbiomes of cesarean-delivered babies, as well as between maternal vagina and the gut microbiomes of vaginal-delivered babies [34]. In disagreement with previous studies, a recent work from Chu et al. reported that even if the infant microbial composition was influenced by the delivery mode at birth, these differences were not present at 6 weeks of age. Interestingly, they found that, unlike the other neonatal body sites, the meconium microbiota at birth is not influenced by the mode of delivery and speculated that the microbial transmission to the fetus may begin at a very early gestational age [35].

Infants’ feeding mode is another main determinant of the microbiome composition [30]. While it is expected that differences occur between breast- and formula-fed babies, we have also to consider that the human milk is not sterile, *Staphylococcus*, *Streptococcus*, and *Pseudomonas* being its most represented bacteria [30]. In addition, the microbial composition of the maternal milk itself is influenced by several factors, such as diet, delivery mode, time post-partum, maternal age, metabolic status, hormone status, and previous medical interventions [30]. Despite these differences, Hunt et al. revealed that a core human milk microbiome is present, and that inter-women variability is higher than intra-woman variability [36].

Considering these factors all together, the so-called first 1000 days of life, from conception until two years of age, are critical for the development of the fetus and infant and can modify the risk profile for diseases onset later in life. After the age of two–three years, the gut microbiome becomes more stable and similar to that of adulthood [2,24,25,30,31,32].

The adult gut microbiome is featured by increased microbial richness and complexity as a consequence of the gradual development of the gut [37]. It is commonly stated that the gut microbial composition is almost similar across adult healthy individuals, because of the development of a core community of microbes that ensure a common, microbial-dependent, functional capability and protect from exogenous insults [37]. Interestingly, this shared microbial composition is present at phylum level, but not at lower phylogenetic levels: a great variability (both qualitative and quantitative) has been described at species and strain levels, suggesting that the gut microbial composition is unique in each individual, even if the microbial functions as a whole result the same [15]. Despite its stability, a number of factors are known to be able to affect the adult gut microbiome, including host genetics, diet, climate, travel, hormonal status, physical activity, and lifestyle [2,15,37]. To solve this discrepancy, it has been recently supposed that the human adult gut microbiome may have two components: one, the core microbiome, almost stable and hardly influenced by external stimuli, and the other, more dynamic and able to change quickly, responsible for microbial plasticity in different conditions and, as a consequence, susceptible to environmental or lifestyle changes [37].

Understanding the role of the gut microbiome is crucial to elucidate human host physiology, since there is growing evidence that it is involved in healthy status acquisition and maintenance [2,15,37,38]. Indeed, even if microbes have been described everywhere in humans, the majority of our microbiota lives in the gut where it is involved in multiple functions, including (but not limited to) the extraction of nutrients from indigestible components of food, the synthesis of essential vitamins and amino acids, the detoxification of potentially harmful molecules, and the defense against pathogens [2,15]. In particular, the gut microbiome has been linked to human physiology through the metabolism of bile acids, choline, and short-chain fatty acids (thus influencing the immune system development) [39]. In addition, it plays a role in gut mucosal permeability, immune system development and self-recognition, energy expenditure, hormonal status, and gut–brain signaling [40]. As a consequence, it is easy to speculate that microbial alterations, or dysbiosis, able to impair these essential functions, may alter human physiology and affect human health status. Accordingly, alterations of the gut microbiome have been related to a number of diseases, both gastrointestinal and non-gastrointestinal, so much so that a provocative report has recently proposed that gut microbiota may be at the intersection of everything [41]. In the attempt to try to generalize knowledge regarding the associations between human gut microbiome and diseases, Duvallet et al. recently carried out a meta-analysis of 28 published gut microbiome studies covering 10 different diseases [42]. This study highlights that, while some diseases are featured by the presence of potentially pathogenic microbes, others are related to the reduction of helpful microbes. Interestingly, these associations seem to be a common pathogenetic mechanism, rather than disease-specific [42].

The challenge of future research in this field will be to assess whether these diseases-related alterations are bystanders, consequences of pathological conditions, or may play a role as triggers or risk factors for specific diseases. In this context, the “common ground hypothesis” has been also conceived to try to establish if the observed gut microbiota dysbiosis is a cause or a consequence of complex, multifactorial diseases. This hypothesis is based on the observation that several factors (endogenous, exogenous and, more often, a combination of them) may affect the gut mucosa inducing permeability alterations and inflammation, also by promoting gut microbiota alterations. In individuals genetically predisposed to a specific disease, such alterations may drive the colonization of the gut by opportunistic and pathobiont microbes that are responsible for local and systemic pathogenic functional modifications contributing to the disease [15].

Finally, it has to be noticed that, even if the term microbiota refers to all the microbes present in an environment, our knowledge on the human microbiota is largely limited to its bacterial fraction, while less is known about other microbial kingdoms (i.e., fungi, archaea, and viruses), their contribution to human health and disease being, actually, largely underestimated. Recent studies, focused on the characterization of fungal communities, have shown that also fungi influence human host physiology and can be modified by different stimuli, including illness [43]. In particular, the human mycobiota seems to play a role in human immunity development and host responses at mucosal level [43]. Similarly, *Archaea* have been indicated as key elements in gut metabolic functions, and modifications in their composition have been related to some diseases [44]. Finally, even viruses could be our symbiotic inhabitants and contribute to our health and disease status [45,46,47]. Therefore, our comprehension of human physiology (and, consequently, of human pathology) must take into account also these aspects and how these microbial groups interact with one another and with the human host. More efforts and future studies are required to address these issues.

## 3. Data Generation, Bioinformatic Issues, and Challenges in Metagenomic Studies

The rapid development of NGS technologies has prompted the study of human microbiome at DNA (16S rRNA and Internal Transcribed Spacers—ITS—sequencing, and shotgun sequencing) and RNA level (metatranscriptomics). Despite the great progresses in this field, technical issues still exist mainly related to the need of continuously updated databases, specific bioinformatic tools, and functional correlations. 

In fact, since sequencing technologies have rapidly increased their throughput and reduced the cost of sequencing/base, a great amount of data is piling up imposing a careful evaluation regarding their use, analysis, and storage. Below, the main bioinformatic tools today available for conducting a microbiome study are briefly reported (Table 1) [48].

For a description of features and issues related to molecular, NGS-based analyses, refer to specific papers on these topics [2,49].

### 3.1. 16S Rrnas and ITS Sequencing

Bacterial 16S ribosomal RNA (rRNA) gene analysis allows the fast characterization of the microbial composition of specific environments. The principle of this approach is that the 16S rRNA gene is a bacterial-specific gene with hyper-variable regions in its sequence that are useful for phylogenetic purposes. Specific bioinformatic tools have been developed for the analysis of 16S rRNA sequences including different functions, like taxonomic assignment, diversity analysis, and functional prediction (Table 1).

Taxonomic assignment is based on the comparison of clustered reads with specific databases of known 16S sequences, i.e., Ribosomal Database Project [50], Greengenes [51], and Silva [52]. To date, different approaches have been optimized for reads clustering (Table 1). Some of these pipelines are based on sequence homology for bacterial identification. In brief, sequence reads obtained in experimental settings are aligned to 16S rRNAs specific databases to assign them to the most similar species. Consequently, the limitation of these approaches is that only the bacterial species annotated in the above-mentioned databases can be identified. Thus, unknown bacteria, corresponding to not aligned reads, are not identified. Greengenes is a specifically designed database of full-length 16S rRNA genes that allows taxonomic identification based on de novo tree inference; however, many reads are not classified [51].

Another possible approach, able to overcome this limitation, is the Operational Taxonomic Units (OTUs) clustering based on the comparison of sequences using different algorithms based on sequence length or pairwise alignment [53,54]. This is the reason why some popular tools for metagenomics, as QIIME (Quantitative Insights Into Microbial Ecology), integrate a combinatorial approach for OTUs clustering [78]. First, sequence reads are aligned against the reference databases for taxonomic assignment. Then, not assigned reads are clustered, based on their sequence similarity to each other. In this way, the taxonomic assignment is more efficient and should avoid the lack of information due to bacterial databases limits. To date, the OTUs clustering has been largely used for 16S analysis; however, its biological relevance has been recently questioned. In fact, even if the clustering of reads allows to minimize the effects of sequencing errors, this operation may affect real phylogenetic diversity, because very similar taxa may be not correctly discriminated. Consequently, alternative strategies, namely sub-OTUs methods, are becoming available. Divisive Amplicon Denoising Algorithm 2 (DADA2) is an open-source algorithm implemented in R, which uses a statistical inference to correct amplicon errors [55]. This package includes all the analytical steps required for amplicon analysis and, by portioning the reads through a denoising algorithm based on the Illumina error model, allows a more accurate bacterial communities resolution with the potential to achieve strain-level information [55]. Similarly, UNOISE2, uses another algorithm optimized for denoising Illumina amplicon reads, which allows reads clustering in zero-radius OTUs (ZOTUs), i.e., OTUs with an identity higher than the conventional 97% [56]. Finally, Deblur promises the resolution of taxa with just one different bp, by using error profiles to infer putative error-free reads [57].

After taxonomic assignment, bacterial communities are compared to highlight significant differences among groups (i.e., patients versus healthy subjects). Actually, one of the most used approaches for this purpose is UniFrac [58]. Unifrac allows communities comparison by measuring the phylogenetic distance between datasets of taxa and implements different tests, such as *p* test and Principal Coordinates Analysis (PCoA) (Table 1). QIIME integrates also these functions [78].

16S rRNA gene sequence analysis provides a rapid and effective way to analyze the entire content of a specific microbial community. Even if it provides taxonomic content and communities comparison and similarity analyses, it does not allow functional evaluations. Recently, specific pipelines are becoming available to infer specific metabolic functions that could be related to a microbial community, suggesting pathways that may be over- or underexpressed in different analyzed samples (Table 1) [59,60,61]. PICRUSt (Phylogenetic Investigation of Communities by Reconstruction of Unobserved States) tool uses 16S rRNA information to infer the functional composition of a metagenome by predicting which gene families are present and their relative abundances [59]. Even if these imputed data need to be experimentally validated, they could provide a primary hypothesis on possible functional alterations associated to a specific disease-related dysbiosis.

Similar issues also concern fungal analysis. In this case, Internal Transcribed Spacers (ITSs), located within the ribosomal gene operon and specific databases, are used as tags for the taxonomic assignment [49].

It has to be noticed that, currently, most of our knowledge related to archaeome is derived from bacterial 16S rRNA studies as a kind of incidental finding [44]. Since this bacterial-targeted approach is not able to detect all the spectrum of *Archaea*, methodological issues have largely limited archaeome qualitative and quantitative characterization [44]. To overcome these limitations, Koskinen et al. proposed a Polymerase Chain Reaction (PCR)-based protocol specifically designed for an in-depth study of the archaeome and assessed the DADA2 pipeline as the most efficient for this kind of studies [44]. These results may be the basis for future investigations specifically designed to study the human archaeome with the aim to characterize it in healt and disease status, unveil its physiological role, and verify a possible contribution of *Archaea* to pathological mechanisms.

No similar approaches are available for other microbial classes (such as viruses), and, also, specific databases are still poor, so that they are usually not taken into account, their contribution to health and disease status being, consequently, actually underestimated.

### 3.2. Shotgun Sequencing

To overcome 16S rRNA genes sequencing limitations, a shotgun strategy, able to analyze the entire genomic content of a community, can be used. The molecular approach is similar to that used for the analysis of a single bacterial genome [79,80], but in this case sequences from both the host and all the microbes (bacteria, archaea, fungi, and viruses) present in the studied environment are obtained. Consequently, the taxonomic assignment requires highly performing assembly tools and updated databases. In fact, in addition to the assembly issues related to NGS (Next Generation Sequencing) reads length, in metagenomic studies we have to consider that we want to assemble a multitude of different genomes that are present in different proportions within the studied community.

Specific assemblers have been developed for shotgun metagenomics (Table 1), such as SOAPdenovo [62], Velvet [63], MetaVelvet [64], Meta-IBDA [65], Genovo [66], Bambus2 [67], and Ray-Meta [68]. Currently, there is no unique consensus regarding the best assembly strategy, since it has been noticed that the assembler performances depend on both biological and technical factors, and different assemblers work better on different datasets [81,82].

Indeed, while, when assembling a single genome, it is possible to use sequencing coverage to identify repeat regions and sequencing errors, in the assembly of a metagenome the sequencing coverage is a consequence of the abundance of each genome within the analyzed community. Thus, the assembler should be able to obtain long contigs for highly represented genomes, without losing the low represented ones [82]. It has to be noticed that, since human and bacterial DNAs are expected to be the most represented in metagenomic datasets, the assembly of poorly represented microbes may result very hard and could generate a loss of information. Consequently, a high sequencing coverage is required to be sure to obtain informative reads representing the entire community, and computational time and space are required to carry out the assembly [82].

In addition, many of the bioinformatic tools currently available for shotgun metagenomics do not allow efficient viruses estimation. In this context, ViromeScan is an open-source tool, specifically developed to analyze virome taxonomy and relative abundance directly from shotgun metagenomic data [69]. ViromeScan is able to enrich viral reads from a metagenomic dataset and map them on a hierarchical and customizable viral database; however, a further implementation with an assembly step is required to allow the detection of new viruses, currently not considered in this analysis [69].

Another difficulty of the assembly step is represented by the possibility to have in the same metagenome two strains of the same bacterial species, whose genomes may differ for few nucleotides. To address this issue, specific pipelines for strain-level analysis are becoming available [82]. In this context, Scholz et al. designed PanPhlAn (pangenome-based phylogenomic analysis), an open-source tool able to obtain strain-level resolution from metagenomic data, also including novel strains identification [70]. Considering all the above, a prudential strategy may be the use of different assembly pipelines, even if costs and time may increase.

Shotgun approaches also allow a functional analysis; to avoid biases introduced during the assembly, some pipelines have been optimized to use unassembled reads for functional prediction. Briefly, a functional analysis is based on genes prediction to infer their probable functions. Different methods, such as FragGeneScan [71], MetaGeneMark [72], and Glimmer-MG [73], have been developed and optimized for this aim. Once the genes have been identified, specific databases can be used for functional predictions, such as IMG database [74], MetaRef [75], dbCAN [76], and HUMAnN [77] (Table 1).

Since the data obtained with this shotgun approach are more complete than those from simple 16S rRNAs or ITS (Internal Transcribed Spacer) analyses, it is conceivable that, as the cost of sequencing decreases, the use of shotgun metagenomics will increase. Even if some biases can be present related to sample collection and storage, DNA extraction procedures, and PCR amplification errors, the shotgun metagenomics offers a comprehensive view of genomes of all the microbes that compose the microbiota of interest. The shotgun metagenomic approach, however, requires high sequence coverage so that taxa in small proportion are represented in the data. Consequently, a high coverage can increase sequencing costs, and the large amount of data that is generated requires additional data storage and specialized analysis pipelines to efficiently catch medical-sensitive information.

### 3.3. Metatranscriptomics

Metatranscriptomics is the analysis of the whole transcriptome of a microbial community. Thus, it provides insight into gene expression and possible functions of human microbiomes in a specific organ or tissue and in a specific condition [83]. In addition to the technical issues related to RNA samples extraction, quality, and purification, comprehensive bioinformatic tools are not still available, and research groups use different in-house developed pipelines. Recently, Westrich et al. described a novel, publicly available pipeline designed ad hoc for metatrascriptomic datasets analysis and gave also useful indications for obtaining good quality data for metatranscriptomics from stool samples [84]. Similarly, Jiang et al. tested their own pipeline on data from different microbiomes showing how, by integrating taxonomic assignment and functional data, it is possible to infer the contribution of specific taxa [85].

Once data analysis will be further optimized and standardized, it is conceivable to suppose that metatranscriptomics may become a very popular approach offering a previously unthinkable way to correlate the presence of specific microbes to specific biochemical and metabolic functions, alterations, or both.

## 4. Conclusions

Metagenomic studies are highlighting that quantitative and/or qualitative microbial alterations occur in an increasing number of diseases. Since the microbiome appears as an actionable target for the development of both novel diagnostic tests and possible targeted therapies, a growing interest has been observed in this topic. This enthusiasm was tempered by the fact that most of the already published studies on the human gut microbiome just report an association with no data regarding functional effects and the possible role on diseases onset or development. The further development and optimization of novel tools for data analysis, integration, correlation, and visualization will allow in a near future to fill in this gap. In this way, it will be possible to understand the functional, mutually beneficial relationship between humans and their gut microbiome during aging and, consequently, its role in the acquisition and maintenance of a healthy status.

## Figures and Tables

**Table 1:** Bioinformatic tools for metagenomic datasets analyses.

Function	Pipeline	Reference
16S Databases	Ribosomal Database Project	(Cole et al., 2014) [50]
	Greengenes	(DeSantis et al., 2006) [51]
	Silva	(Quast et al., 2013) [52]
OTUs clustering	Cd-hit	(Li et al., 2006) [53]
	UCLUST	(Edgar et al., 2010) [54]
Sub-OTU methods	DADA2	(Callahan et al., 2016) [55]
	UNOISE2	(Edgar, 2016) [56]
	Deblur	(Amir et al., 2017) [57]
Communities diversity comparison	UniFrac	(Lozupone et al., 2006) [58]
Functional profiles prediction	PICRUSt	(Langille et al., 2013) [59]
	Piphillin	(Iwai et al., 2016) [60]
	Tax4Fun	(Aßhauer et al., 2015) [61]
Metagenome assembly	SOAPdenovo	(Li et al., 2010) [62]
	Velvet	(Zerbino et al., 2008) [63]
	MetaVelvet	(Namiki et al., 2012) [64]
	Meta-IBDA	(Peng et al., 2011) [65]
	Genovo	(Laserson et al., 2011) [66]
	Bambus2	(Koren et al., 2011) [67]
	Ray-Meta	(Boisvert et al., 2012) [68]
	ViromeScan	(Rampelli et al., 2016) [69]
	PanPhlAn	(Scholz et al., 2016) [70]
Gene identification	FragGeneScan	(Rho et al., 2010) [71]
	MetaGeneMark	(Zhu et al., 2010) [72]
	Glimmer-MG	(Kelley et al., 2012) [73]
Functional assignment	IMG database	(Markowitz et al., 2014) [74]
	MetaRef	(Huang et al., 2014) [75]
	dbCAN	(Yin et al., 2012) [76]
	HUMAnN	(Abubucker et al., 2012) [77]

## Abbreviations

NGS	Next-Generation Sequencing
rRNA	ribosomal RNA
OTUs	Operational Taxonomic Units
QIIME	Quantitative Insights Into Microbial Ecology
DADA2	Divisive Amplicon Denoising Algorithm 2
ZOTUs	zero-radius OTUs
Bp	base pair
PCoA	Principal Coordinates Analysis
PICRUSt	Phylogenetic Investigation of Communities by Reconstruction of Unobserved States
ITSs	Internal Transcribed Spacers
PCR	Polymerase Chain Reaction
PanPhlAn	pangenome-based phylogenomic analysis
